# Cross-Sectional Study of Occlusal Loading and Periodontal Status of Teeth with Deflective Occlusal Contacts

**DOI:** 10.3390/bioengineering12070766

**Published:** 2025-07-16

**Authors:** Ximena Anca Nicolae, Elena Preoteasa, Catalina Murariu Magureanu, Cristina Teodora Preoteasa

**Affiliations:** 1 Department of Dental Prosthodontics, Faculty of Dental Medicine, “Carol Davila” University of Medicine and Pharmacy, 041313 Bucharest, Romaniacatalina.magureanu.murariu@umfcd.ro (C.M.M.); 2 Department of Scientific Research Methodology and Ergonomics, Faculty of Dental Medicine, “Carol Davila” University of Medicine and Pharmacy, 041313 Bucharest, Romania

**Keywords:** digital occlusal analysis, nonfunctional occlusal contact, occlusal trauma, periodontal disease

## Abstract

Aim: To evaluate whether maximum occlusal loading and periodontal status are different between teeth presenting deflective occlusal contacts and those without such contacts, specifically adjacent and homologous teeth. Method: A cross-sectional study was conducted using OccluSense to detect deflective contacts and quantify occlusal load per tooth. For group comparisons, the Kruskal–Wallis, Friedman, Cochran’s Q, and chi-squared tests were used. Results: A total of 493 teeth with deflective contacts were compared to 473 adjacent (first control group) and 457 homologous teeth (second control group). Teeth with deflective contacts showed significantly higher occlusal loading (mean value: 208) than adjacent (72) and homologous teeth (97) (*p* < 0.05). They also exhibited more advanced periodontal damage, including deeper probing depths, greater gingival recession, alveolar bone loss, and a wider periodontal ligament space. Deflective contacts in centric relation were more strongly linked to periodontal deterioration than those in protrusive or lateral mandibular movements, despite similar occlusal forces. Conclusions: Within this study’s limitations, deflective occlusal contacts are associated with increased occlusal forces and more severe periodontal damage, suggesting a biomechanical factor in periodontal disease progression.

## 1. Introduction

Periodontal disease is a serious public health problem and one of the most prevalent conditions worldwide. It is characterized by inflammation and destruction of the periodontal tissues that support the teeth [1]. Periodontitis affects approximately 50% of the world’s adult population, and its prevalence and severity increase with age. Occlusal trauma is a condition that occurs when excessive forces are applied to the teeth. These forces can be caused by a variety of factors, which are broadly divided into the categories of precipitating factors and predisposing factors [2,3]. The precipitating factors are related to the force descriptors such as magnitude, direction, duration, and frequency [3]. The predisposing factors are also divided into intrinsic factors, defining the orientation force of the long axis of the root, morphological characteristics of the root or of the alveolar bone, and extrinsic factors such as the presence of dental plaque, periodontal inflammation, teeth loss, presence of iatrogenic factors, bruxism, clenching, tooth migration, TMJ dysfunction [2,3].

The forces exerted on teeth are involved in shaping their form, position, and functions of the periodontal ligaments, which must be mentioned because they also influence periodontal healing processes [4,5]. The response of periodontal tissue to traumatic forces occurs in three stages: active tissue destruction, tissue adaptation, and tissue remodeling [6,7,8,9]. Trauma from occlusion occurs when the intensity of the forces applied to the periodontium exceeds the adaptive capacity of the periodontal supportive tissues or if the periodontium has a pre-existing periodontal condition that impairs its ability to adapt to physiological occlusal forces [9]. Regardless of the situation, the changes produced are similar, namely: in the presence of occlusal trauma, alveolar bone resorption, a significant reduction in the number of periodontal ligaments/root volume unit, deepening of periodontal pockets, the appearance of gingival recession, tooth migration and, not least pathological tooth mobility, all of which have a major impact on the functionality of teeth [4].

In periodontics, the prevention, treatment, and maintenance of the integrity of the periodontal support apparatus are essential for ensuring long-term periodontal health stability. From this perspective, a variety of favorable factors can influence the periodontal status and, more importantly, the treatment directions. Therefore, the goal of periodontal disease treatment is to properly identify and control these factors.

An association between the occlusal trauma and periodontal status is suggested by previous research of different types, e.g., in silico, animal, and human studies [10,11,12,13,14,15]. More severe periodontal damage was identified in cases of occlusal trauma in some research [10,11,13,14,15], but not in others [16,17]. Generally, research on this topic is relatively limited, and findings are not uniform, probably due to the complex nature of the relationship and difficulties in its investigation. Through advances in the dental field, specifically referring to the digital methods for registrations and analysis of dental occlusion, data on this topic, with great importance from a clinical point of view, could be obtained. Studies comparing occlusal records made by classical versus modern methods have shown that the latter offers a higher degree of accuracy, particularly by reducing the risk of subjectivity in the interpretation of occlusal records [14,15,18,19,20,21].

This study aims to assess whether maximum occlusal loading and periodontal status differ significantly between teeth with and without deflective occlusal contacts. Specifically, the analysis involves comparisons of teeth with deflective occlusal contact with adjacent and homologous control teeth. Secondly, it aims to assess whether maximum occlusal loading and periodontal status differ regarding the type of occlusal contact, i.e., premature contacts, protrusive interference, and lateral movement interference.

## 2. Materials and Methods

This study was approved by the Research Ethics Committee of Carol Davila University of Medicine and Pharmacy (protocol code PO-35-F-03 and date of approval: 14 March 2025).

Prior to inclusion in this study, participants were informed about the research and provided written consent by signing the informed consent form.

A cross-sectional study was conducted on a convenience sample of patients enrolled consecutively, as they met the following eligibility criteria and agreed to participate in the research. Adult patients with primary and/or secondary occlusion disorders, identified based on a digital occlusal examination, were included in this study. Additional inclusion criteria were a minimum age of 18 years, patients with at least 10 pairs of occlusal units, and patients who had undergone a panoramic radiograph within the last 6 months. Exclusion criteria were patients who had received occlusal adjustments or periodontal treatments within the past 12 months, excluding visits for dental prophylaxis; patients with systemic conditions known to be associated with periodontal disease, such as diabetes, hypertension, blood disorders, kidney disease, or a history of radiotherapy and chemotherapy; pregnant women; and those who refused or were unable to cooperate. After analyzing previous research on the topic [22,23], a sample size of 50 participants was targeted. They were included based on availability and convenience during routine clinical examinations.

General data, including age and gender, as well as information regarding dental, prosthetic, periodontal, and occlusal status, were recorded. Data were collected through interviews, clinical examinations, and paraclinical examinations using a digital occlusal analyzer (OccluSense; Dr. Jean Bausch GmbH & Co. KG, Cologne, Germany) and analysis of orthopantomograms.

**Figure 1:**
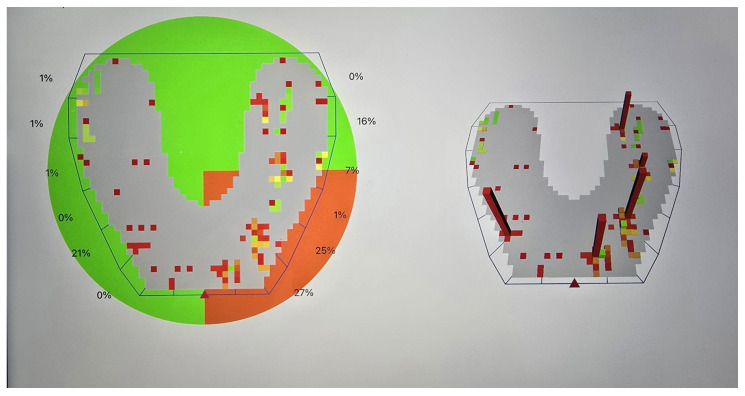
Centric relation—occlusion map obtained by OccluSense device (Dr. Jean Bausch GmbH & Co. KG). A color scale is used to mark a large pressure change between one contact and its adjacent contact points, varying from green (shows a small pressure change) to red (a large pressure change).

**Figure 2:**
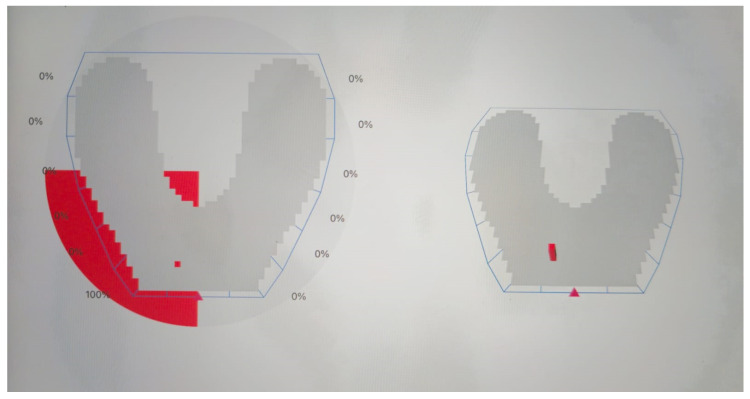
Protrusive movement—occlusion map obtained by OccluSense device (Dr. Jean Bausch GmbH & Co. KG). A color scale is used to mark a large pressure change between one contact and its adjacent contact points, varying from green (shows a small pressure change) to red (a large pressure change).

**Figure 3:**
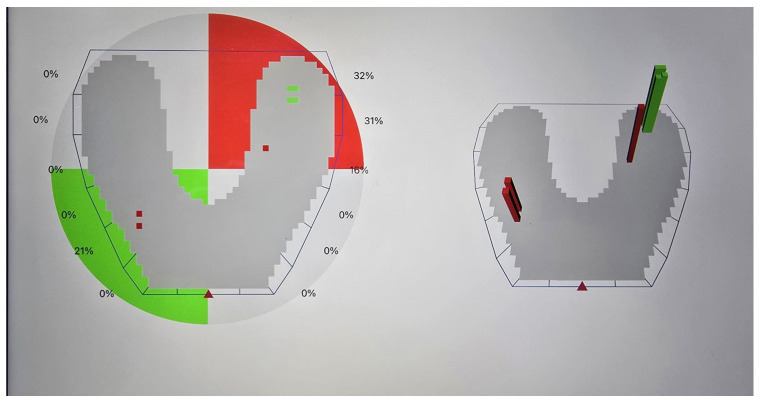
Right lateral movement—occlusion map obtained by OccluSense device (Dr. Jean Bausch GmbH & Co. KG). A color scale is used to mark a large pressure change between one contact and its adjacent contact points, varying from green (shows a small pressure change) to red (a large pressure change).

Deflective occlusal contacts are defined in this study as nonfunctional occlusal contacts that cause a deviation in the normal path of mandibular closure [24]. These may include premature contacts occurring in centric relation and interferences during lateral or protrusive movements, resulting in altered force distribution and potential periodontal damage (Figure 1, Figure 2 and Figure 3).

The occlusal examination aimed to identify deflective occlusal contacts, categorized into premature contacts and interferences. Premature contacts are present during the centric path of mandibular closure, just before reaching the position of maximal intercuspal, and when the mandible is deflected. The occlusal interferences are nonfunctional occlusal contacts occurring during left/right lateral mandibular movements or protrusion. For each tooth with a deflective occlusal contact, regardless of its type, data were recorded for two additional teeth used for comparison: an adjacent tooth (the adjacent tooth mesial to the affected tooth, and if the mesial tooth was missing, data were recorded for the distal tooth, if present) (1); and the homologous tooth, if present (2). For these three teeth (the tooth with the nonfunctional occlusal contact, the adjacent tooth, and the homologous tooth), the same data were recorded, i.e., dental, prosthetic, periodontal status, and details regarding the characteristics of the occlusal contact.

The OccluSense device incorporates a sensor with 256 pressure levels, designed for single-use applications, that records occlusal force distribution across the dental arch. The data are transmitted wirelessly to an iPad application (Apple Inc., Cupertino, CA, USA) that visualizes the forces in both 2D and 3D formats, with quantifiable intensity expressed as a percentage.

For the occlusal examination, the OccluSense device (Bausch) was used, with a frequency of 100 Hz and a recording duration of 10 s. The sensor of the device has a thickness of 60 µm, and its edges are embedded in a cardboard support designed to attach to the device’s handle. Both sides of the sensor are covered with red articulation paper, which helps to imprint the occlusal contact areas on the opposing dental surfaces. The sensor is single-use, and through its 256 pressure points, it records the distribution of occlusal forces as a percentage, as well as the differences in the intensity of occlusal pressures on the occlusal surfaces. These data can be identified using the associated application on an IPAD tablet, with visualization available in 2D/3D. After the occlusal analysis, for each of the teeth, i.e., the tooth with the nonfunctional occlusal contact, the adjacent tooth, and the homologous tooth, the maximum pressure level recorded at the tooth level was noted.

**Figure 4:**
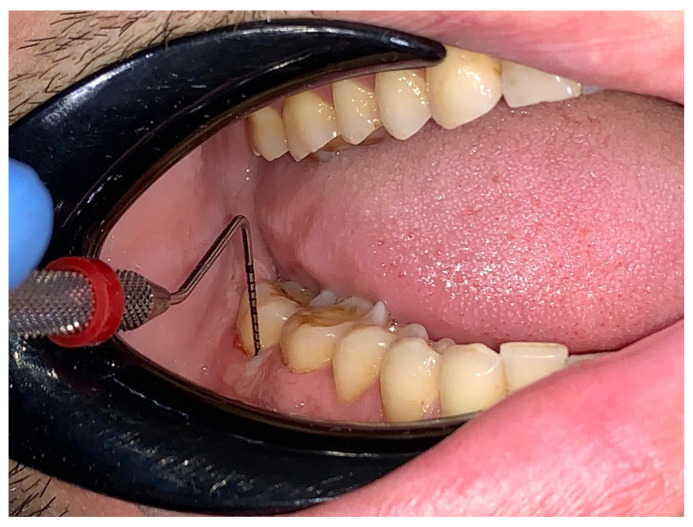
Probing depth examination.

**Figure 5:**
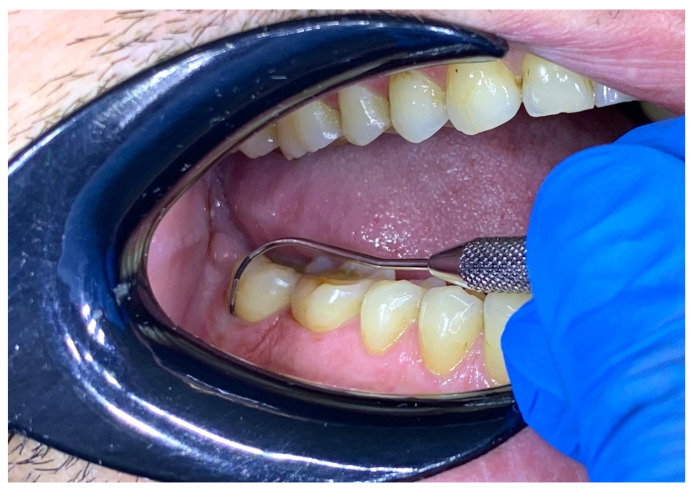
Furcation involvement examination.

Clinical periodontal examination was performed at six sites per tooth using the UNC15 periodontal probe (Hu-Friedy, Chicago, IL, USA) to assess probing depth (Figure 4) and gingival recession: mesio-buccal, mid-buccal, disto-buccal, mesio-lingual, mid-lingual, and disto-lingual. Nabers probe (Hu-Friedy, Chicago, IL, USA) was used to evaluate furcation involvement (Figure 5). Representative images of probing are included to illustrate the assessment methodology. The probing depth of the gingival sulcus/periodontal pocket and gingival recession were assessed per tooth/implant, with the maximum values recorded in millimeters, starting from the gingival margin to the deepest probing point in gingival sulcus, for the probing depth and starting from the cemento-enamel junction to the gingival margin, for the gingival recession. Furcation involvement was registered according to Hamp’s classification [25]. Dental mobility was recorded according to Miller’s classification [26], while the degree of dental wear was assessed using the Smith and Knight classification [27].

**Figure 6:**
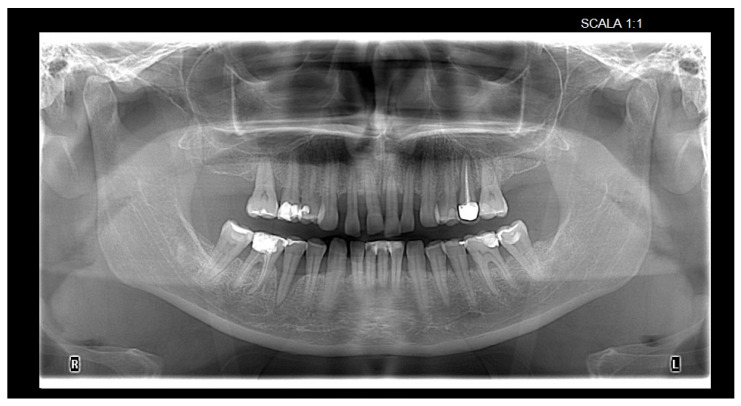
Orthopantomogram evaluation.

Radiographic examination of orthopantomograms was used to evaluate proximal bone loss (Figure 6). Reference points for the measurements were the cemento-enamel junction, root apex, alveolar crest, and the deepest point of the infrabony defect.

One researcher performed a full examination of all the participants, i.e., X.A.N.

Data analysis was conducted using SPSS Statistics software, version 22. To compare groups, the Friedman test, Kruskal–Wallis test, chi-squared test, and Cochran’s Q test were used, depending on the type of data, either numerical or categorical, and whether the groups being compared were dependent or independent. The non-parametric Kruskal–Wallis and Friedman statistical tests were used due to data not being normally distributed. For comparing groups with categorical data, the chi-squared and Cochran’s Q tests were applied. The statistical significance threshold was set at *p* < 0.05. Reporting of *p*-values for pairwise comparisons was performed using the Bonferroni correction.

## 3. Results

Of the 52 participants, 32 were female and 20 were male, with a mean age of 31.35 years. Correspondingly, 493 teeth with deflective occlusal contacts, 473 adjacent teeth (control group 1), and 457 homologous teeth (control group 2) were analyzed.

Teeth with deflective occlusal contacts recorded a statistically significantly higher maximum occlusal loading, as well as signs of greater severity of marginal periodontium impairment (with respect to periodontal probing depth, gingival recession, alveolar bone loss assessed radiographically, and a significantly more frequent widening of the periodontal space). Additionally, teeth with deflective occlusal contacts exhibited a statistically significantly higher frequency of dental migration and a greater degree of tooth wear (Table 1).

Occlusal interferences were recorded more frequently than premature contacts. From occlusal interferences, protrusion interferences were most frequently encountered. Right lateral movement interferences were more frequently encountered than left lateral movement interferences (Table 2).

Regarding the type of deflective occlusal contact observed (premature contact or interference), the maximum occlusal loading recorded similar values; however, a statistically significant difference was observed between deflective occlusal contacts in protrusion and lateral mandibular movements. Teeth with premature contacts exhibited significantly more severe signs of periodontal impairment compared to teeth with protrusive or lateral interferences, concerning periodontal probing depth, gingival recession, tooth mobility, and alveolar bone loss. Teeth with premature contacts also more frequently exhibited abfraction lesions, tooth migration, and widening of the periodontal space as seen radiologically, with the differences between groups being statistically significant (Table 3).

## 4. Discussion

The role of occlusal trauma in the etiology of periodontal diseases has been a topic of debate since the early 20th century. Initial studies were conducted on laboratory animals and human cadavers, and the findings suggested a potential correlation between the presence of occlusal trauma and the development of bone defects, and ischemia within the periodontal ligament has been identified as the primary triggering factor [10,16,17].

Physiological occlusion refers to a state in which the distribution of occlusal forces upon a tooth is in equilibrium, maintaining a stable relationship between the tooth and its supporting periodontal structures [28]. In this balanced state, occlusal pressure is counteracted by the resistance of the periodontal apparatus, preventing any structural disruption [28].

Traumatic occlusion, by contrast, occurs when excessive or misdirected occlusal forces lead to damage within the periodontium [28]. Acute trauma may result from sudden, high-impact forces—such as biting on a hard object—or from improperly designed restorations or prostheses that alter the direction or magnitude of occlusal loading [28].

Chronic trauma typically develops over time, resulting from progressive changes in the occlusal scheme [28]. These may include tooth wear, drifting, extrusion, or the presence of parafunctional habits such as bruxism and clenching [28]. Unlike acute trauma, chronic trauma emerges gradually and is not necessarily preceded by an acute event [28].

Primary traumatic occlusal forces occur when abnormal forces act on a periodontium that is otherwise healthy [28]. In contrast, secondary traumatic occlusal forces involve either normal or excessive occlusal forces applied to a periodontium that is already compromised, typically by significant loss of alveolar bone support [28].

A critical distinction exists between physiological and pathological occlusal forces. Physiological forces are those that remain within the adaptive capacity of the periodontium, contributing to the maintenance of periodontal tissue integrity during normal function [29]. Pathological forces, however, exceed this adaptive threshold, potentially resulting in injury to the periodontal ligament, widening of the periodontal space, and progressive alveolar bone loss [29]. The significantly higher occlusal loading recorded in teeth with deflective contacts in our study suggests a transition from physiological to pathological force dynamics, particularly when such contacts persist in centric relation. This highlights the need for the timely identification and correction of these forces to prevent biomechanical overloading and subsequent periodontal deterioration.

Alveolar bone homeostasis is maintained through a dynamic balance between bone formation by osteoblasts and resorption by osteoclasts [29]. Physiological forces, such as those generated during mastication and normal occlusal function, enhance blood circulation and metabolic activity within periodontal tissues [29]. Conversely, the absence of functional occlusion can lead to periodontal tissue atrophy and progressive bone resorption [29].

While mild pathological forces—such as those from traumatic occlusion—may induce vertical resorption of the alveolar ridge and increase tooth mobility, they do not typically result in gingival inflammation, pocket formation, or clinical attachment loss. In certain cases, a tooth subjected to sustained pathological loading may shift or tilt toward the side of compression, a compensatory response like orthodontic tooth movement [29].

A definitive diagnosis of occlusal trauma is obtained after histological analysis of the changes that occur at the level of the periodontium, a fact considered inadequate for conducting interventional research on human subjects but proven by animal studies. Thus, we can discuss a presumptive diagnosis of occlusal trauma by associating several clinical and radiographic signs, such as the presence of fremitus, pathological tooth mobility, premature contact, interference in mandibular movements, tooth abrasion, tooth migration, discomfort or pain when chewing, tooth fracture or root fracture, root resorption, widening of the periodontal space, and non-carious cervical lesions. These changes are also common in other oral pathologies, which is why careful occlusal and periodontal analysis is required to obtain the correct diagnosis and highlight a possible correlation between the presence of occlusal pathology and periodontal changes. At the same time, previous research supports the idea that there is insufficient evidence to support that occlusal trauma is linked to the onset or accelerates the progression of periodontal diseases [6,30], especially because the periodontal ligament physiologically adapts to excessive occlusal forces by resorbing alveolar bone, followed by tooth mobility [6]. Thus, the prevailing view is that occlusal trauma may act as a cofactor, playing a role in accelerating the progression of periodontal diseases, and occlusal therapy is an integral part of complex periodontal treatment but does not replace non-surgical periodontal therapy [30,31].

Our study demonstrates, through clinical observation and digital quantification, that deflective occlusal contacts are associated with significantly higher occlusal forces and increased periodontal deterioration compared to adjacent and homologous teeth. These findings are in line with previous clinical observations suggesting that occlusal trauma leads to the disorganization of the periodontium, negatively impacting its local reparative capacities [17]. Additionally, it increases probing depth, which facilitates the spread of inflammation to adjacent areas [32].

Studies on animals have not demonstrated a correlation between occlusal trauma and periodontal disease. However, it is important to emphasize that in animals, mandibular dynamics differ, and the periodontium exhibits a significantly better response to various occlusal stimuli [11,12].

Between the 1960s and 1970s, Glickman and colleagues introduced the concept of occlusal trauma as a destructive factor for periodontal tissues. This process was explained through the spread of gingival inflammatory exudate into the periodontal ligaments, leading to the formation of a combined lesion characterized by the development of infrabony pockets [13,14]. However, the coexistence of these two factors (occlusal and periodontal) without the presence of infrabony defects cannot be ruled out. Additionally, there are cases of suprabony pockets accompanied by horizontal bone resorption where occlusal trauma is also present [15]. The type of periodontal pockets that develop in a patient with periodontal disease as a response to occlusal trauma is correlated with the thickness of the bony wall; the thicker the alveolar bone, the greater the likelihood of infrabony periodontal pockets forming, and vice versa [33]. Additionally, occlusal trauma induces structural changes in the alveolar bone, promoting the transformation of areas with suprabony defects into areas with combined defects [33]. Furthermore, regardless of the type of occlusal trauma, there are no therapeutic differences in terms of periodontal manifestations [18].

Shefter and McFall (1984) [34] investigated the correlation between 4–6 mm periodontal pockets and the presence of deflective occlusal contacts in centric relation, as well as during lateral and protrusive mandibular movements. They found that teeth with premature contacts during protrusion tended to have larger periodontal pockets [34].

Jin and Cao (1992) [35] considered premature contacts in centric relation as well as in the dynamics of mandibular movements in their study. Their results suggested that there is no correlation between these deflective occlusal contacts and increased probing depth. However, they observed that such issues influence the widening of the periodontal space [35].

Follow-up, after at least one year, revealed that teeth without occlusal adjustment had significantly greater probing depths compared to those where the occlusal discrepancies were corrected or compared to teeth without any occlusal problems [36]. This study supports the idea of a potential link between the presence of occlusal interferences and changes in the clinical attachment level [37]. However, a limitation of this study is that the therapeutic options were not randomly assigned to the subjects; the approach was chosen based on the patient’s preferences [37,38].

In 2004, Nunn and Harrel [39] reassessed the role of occlusion in the progression of periodontal disease. They concluded that occlusal trauma is a predisposing factor for the development of periodontal pockets, but no significant differences were found between the types of deflective occlusal contacts and periodontal disease [39]. The results suggested that any type of occlusal discrepancies can lead to an increase in probing depth, on average by 1 mm [39]. At the same time, occlusal adjustments were not associated with a reduction in probing depth, but they did improve the clinical attachment level, on average by approximately 0.4 mm [39]. Our current results support the hypothesis that occlusal overload contributes to periodontal damage, particularly when deflective occlusal contacts occur in centric relation.

Also, in a study conducted by Ishigaki et al. (2006) [40], regarding the impact of occlusal interferences during mastication on tooth mobility, the findings showed that in men, the upper central incisors and the first and second upper premolars exhibited notable differences in mobility between groups with normal chewing patterns compared to those with moderate to severe deviations from normal patterns [40]. In women, tooth mobility differences were observed in incisors, canines, premolars, and molars across the three groups, in both the upper and lower jaws [40].

In a study conducted by Bernhardt et al. (2006) [41], which investigated the association between the presence of occlusal interferences and signs of periodontal involvement in posterior teeth, the results showed that occlusal interferences on the non-working side were correlated with the presence of periodontal pockets and loss of clinical attachment level, on average, an increase of 0.13 mm in periodontal probing depth and an additional loss of 0.14 mm in clinical attachment level were reported in the presence of occlusal interferences on the non-working side [41]. In cases where occlusal interferences were present on both the working and non-working sides, an increase in periodontal probing depth was observed, but without affecting the clinical attachment level [41].

Subsequent studies conducted by Harrel and Nunn, Zhou and Fang, and Rios [31,42,43] have demonstrated an association between the presence of premature contacts or occlusal interferences and the loss of clinical attachment level or degradation of periodontal tissues, particularly in the molar-premolar areas [31,42,43]. For the frontal teeth areas, no statistically significant association was found between the presence of occlusal trauma and the severity of periodontal changes [42,43,44].

This study is novel in its use of digital occlusal force mapping (OccluSense device; Dr. Jean Bausch GmbH & Co. KG) to correlate localized occlusal load variations with corresponding periodontal deterioration at the single-tooth level, a methodological detail not extensively explored in prior human studies.

Our study, conducted on a sample of 52 individuals with an average age of 31.35 years and involving 1423 teeth or implants with deflective occlusal contacts, adjacent teeth, or homologous teeth, showed differences in the intensity of adjacent tooth-to-tooth contacts and periodontal disease manifestations, which could be correlated to one another. The greatest differences in contact intensity were recorded on teeth with deflective occlusal contacts, which also exhibited more severe periodontal disease manifestations compared to adjacent and homologous teeth. This finding is aligned with other authors who have found a significant mean difference in occlusal force between severe, moderate, and mild clinical attachment loss (*p* = 0.000) [45].

It can also be observed that the differences in the intensity of tooth-to-tooth contacts are greater in homologous teeth than in adjacent teeth, with greater periodontal probing depth, widening of the periodontal space, and alveolar bone loss. Although another study found only a correlation between the intensity of occlusal contacts and alveolar bone loss and tooth mobility (*p* < 0.03) [46], adjacent teeth also show some signs of periodontal disease (gingival recession, dental mobility), as well as occlusal trauma features like those of teeth with deflective occlusal contacts (dental wear, abfraction). Regarding this topic, Bozhkova (2016) [47] identified an increase in probing depth as well as a greater loss of clinical attachment level on the non-working side [47]. Increased mobility in adjacent teeth could explain the reduction in the differences in the intensity of adjacent tooth-to-tooth contacts, compared to homologous teeth, at this stage of evaluation. Nalini et al. (2024) [23] were unable to demonstrate that occlusal adjustment combined with non-surgical periodontal treatment has a favorable effect on alveolar bone gain, with the limitation being the short follow-up period of 6 months [23].

Concerning the importance of using digital occlusal analysis devices, this study demonstrated the benefit of detecting deflective occlusal contacts during eccentric mandibular movements, while other studies have shown a greater impact from detecting occlusal discrepancies in centric occlusion [22,48]. This idea is aligned with our findings. Additionally, Deepika (2022) [22] emphasized that among patients with periodontal disease, occlusal disorders were significantly more pronounced in centric relation, protrusion, and right lateral movement in men, while in women, changes were more prevalent in left lateral movement [22].

Study Limitations: This study is limited by its cross-sectional design, which does not allow for causal inference. The sample size, although adequate for initial observations, limits generalizability. Nevertheless, the sample size may not fully represent the broader population, and this limitation is acknowledged. Additionally, imaging and clinical assessments were subject to potential examiner bias despite calibration. The lack of a long-term follow-up also prevents evaluation of disease progression or treatment efficacy. Also, when analyzing the relationship between periodontal status and occlusal loading, confounders linked to occlusal and anatomical particularities should be considered in future research to have a more in-depth perspective on this topic.

## 5. Conclusions

Occlusion and periodontal health appear to be functionally interconnected. Our study demonstrates that teeth with deflective occlusal contacts are subjected to significantly greater occlusal loads and show more advanced periodontal deterioration than adjacent or homologous teeth. Premature contacts are linked to the most severe periodontal findings. These results emphasize the importance of identifying and managing occlusal discrepancies during periodontal assessment and treatment.

The digital evaluation of occlusion using the OccluSense System is a straightforward, user-friendly tool that can provide valuable insights into occlusal loading, helping to explain certain clinical manifestations observed in the periodontal tissues. This occlusal assessment, performed before, during, and after occlusal interventions, can guide and contribute to better diagnosis and treatment plans and achieve better outcomes in dental treatments.

## Figures and Tables

**Table 1:** Comparison of occlusal load and periodontal status in teeth with and without deflective occlusal contact.

Variable	1. Tooth with Deflective Occlusal Contact N = 493	2. Adjacent Tooth N = 473	3. Homologous Tooth N = 457	*p*	*p*_1-2_	*p*_1-3_	*p*_2-3_
**Maximum occlusal loading (mean)**	208	72	97	<0.001 ^1^	<0.001	<0.001	0.026
**Probing depth (mean)**	4.15 mm	3.61 mm	3.7 mm	<0.001 ^1^	<0.001	0.014	0.224
**Gingival recession (mean)**	0.83 mm	0.69 mm	0.65 mm	0.002 ^1^	>0.999	0.081	0.429
**Tooth mobility (mean)**	0.24 mm	0.24 mm	0.19 mm	0.052 ^1^			
**Abfraction (n; %)**	N = 33; 7%	N = 35; 7%	N = 25; 5%	0.199 ^2^			
**Pathologic tooth migration (n, %)**	N = 131; 27%	N = 83; 18%	N = 96; 21%	<0.001 ^2^	<0.001	0.001	0.497
**Tooth wear (mean)**	0.45	0.38	0.36	<0.001 ^1^	0.129	0.099	>0.999
**Alveolar bone loss Rx (mean)**	1.59 mm	1.15 mm	1.25 mm	<0.001 ^1^	0.003	0.037	>0.999
**Widening of periodontal space (n, %)**	N = 52; 11%	N = 30; 7%	N = 31; 7%	0.005 ^2^	0.012	0.018	>0.999

*p*—*p*-value for comparing teeth with deflective occlusal contacts (1), adjacent teeth (2), and homologous teeth (3). *p*_1-2_; *p*_1-3_; *p*_2-3_*—p*-value for pairwise comparison. ^1^ Friedman test. ^2^ Cochran’s Q test. Significance values for pairwise comparisons have been adjusted by the Bonferroni correction.

**Table 2:** Distribution of deflective occlusal contacts.

Deflective Occlusal Contact	N	%
Premature contacts	68	13.8%
Protrusive interferences	212	43.0%
Right lateral movement interferences	117	23.7%
Left lateral movement interferences	96	19.5%
Total	493	100.0%

**Table 3:** Occlusal load, periodontal, and dental status in relation to the type of deflective occlusal contact.

Variable	1. Premature Contact N = 68	2. Protrusive Interference N = 212	3. Lateral Movement Interference N = 213	*p*	*p*_1-2_	*p*_1-3_	*p*_2-3_
**Maximum occlusal loading (mean)**	211	215	206	0.009 ^1^	>0.999	0.620	0.006
**Periodontal probing depth (mean)**	5.07 mm	4.21	3.81	<0.001 ^1^	0.010	<0.001	0.042
**Gingival recession (mean)**	1.59 mm	0.76	0.65	<0.001 ^1^	0.009	<0.001	0.508
**Tooth mobility (mean)**	0.51	0.22	0.17	<0.001 ^1^	0.001	<0.001	>0.999
**Abfraction (n; %)**	N = 15; 22%	N = 13; 6%	N = 5; 2%	<0.001 ^2^			
**Pathologic tooth migration (n, %)**	N = 37; 54%	N = 52; 25%	N = 42; 20%	<0.001 ^2^			
**Tooth wear (mean)**	0.57	0.44	0.42	0.126 ^2^			
**Alveolar bone loss Rx (mean)**	2.99 mm	1.45 mm	1.28 mm	<0.001 ^1^	<0.001	<0.001	>0.999
**Widening of periodontal space (n, %)**	N = 14; 21%	N = 22; 10%	N = 16; 11%	0.009 ^2^			

*p*—*p*-value for comparing teeth with premature contacts (1), protrusive interferences (2), and lateral movement interferences (3). *p*_1-2_; *p*_1-3_; *p*_2-3_—*p*-value for pairwise comparison. ^1^ Kruskal–Wallis test. ^2^ Chi-squared test. Significance values for pairwise comparisons have been adjusted by the Bonferroni correction.

## Data Availability

The original contributions presented in this study are included in this article. Further inquiries can be directed to the corresponding author.
